# Lung epithelial cell-derived IL-25 negatively regulates LPS-induced exosome release from macrophages

**DOI:** 10.1186/s40779-018-0173-6

**Published:** 2018-07-30

**Authors:** Zhi-Gang Li, Melanie J. Scott, Tomasz Brzóska, Prithu Sundd, Yue-Hua Li, Timothy R. Billiar, Mark A. Wilson, Ping Wang, Jie Fan

**Affiliations:** 1 Department of Surgery, University of Pittsburgh School of Medicine, Pittsburgh, PA 15213 USA; 2 Research and Development, Veterans Affairs Pittsburgh Healthcare System, Pittsburgh, PA 15240 USA; 3 Heart, Lung, Blood and Vascular Medicine Institute, University of Pittsburgh, Pittsburgh, PA 15213 USA; 4 McGowan Institute for Regenerative Medicine, University of Pittsburgh, Pittsburgh, PA 15219 USA; 5 The Feinstein Institute for Medical Research, Manhasset, NY 11030 USA

**Keywords:** Acute lung injury, Sepsis, Multiple organ failure, Rab27

## Abstract

**Background:**

Acute lung injury (ALI) is a major component of multiple organ dysfunction syndrome (MODS) following pulmonary and systemic infection. Alveolar macrophages (AMϕ) are at the center of ALI pathogenesis. Emerging evidence has shown that cell-cell interactions in the lungs play an important regulatory role in the development of acute lung inflammation. However, the underneath mechanisms remain poorly addressed. In this study, we explore a novel function of lung epithelial cells (LEPCs) in regulating the release of exosomes from AMϕ following LPS stimulation.

**Methods:**

For the in vivo experiments, C57BL/6 wildtype (WT) mice were treated with lipopolysaccharide (LPS) (2 mg/kg B.W.) in 0.2 ml of saline via intratracheal aerosol administration. Bronchoalveolar lavage fluid was collected at 0–24 h after LPS treatment, and exosomes derived from AMϕ were measured. For the in vitro studies, LEPCs and bone marrow-derived Mϕ (BMDM) were isolated from WT or TLR4^−/−^ mice and were then cocultured in the Transwell™ system. After coculture for 0–24 h, the BMDM and supernatant were harvested for the measurement of exosomes and cytokines.

**Results:**

We demonstrate that LPS induces macrophages (Mϕ) to release exosomes, which are then internalized by neighboring Mϕ to promote TNF-α expression. The secreted interleukin (IL)-25 from LEPCs downregulates Rab27a and Rab27b expression in Mϕ, resulting in suppressed exosome release and thereby attenuating exosome-induced TNF-α expression and secretion.

**Conclusion:**

These findings reveal a previously unidentified crosstalk pathway between LEPCs and Mϕ that negatively regulates the inflammatory responses of Mϕ to LPS. Modulating IL-25 signaling and targeting exosome release may present a new therapeutic strategy for the treatment of ALI.

## Background

Acute lung injury (ALI) is a major component of multiple organ dysfunction syndrome (MODS) following sepsis [1, 2]. However, information about the factors that predispose septic patients to MODS or ALI remains poor, and this paucity of knowledge contributes to high mortality rates and a lack of effective treatments [2–4]. Emerging evidence suggests an important role for cell-cell interaction in the regulation of ALI progression [5–7]. For instance, we have reported a role for hemorrhagic shock-activated polymorphonuclear neutrophils (PMN) that have migrated to the lung in counteracting the effects of anti-inflammatory NOD2**-**induced autophagy of alveolar macrophages (AMϕ) [6]. The current study investigates important interactions between lung epithelial cells (LEPCs) and AMϕ.

Residential AMϕ serve as the front line of cellular defense in the recognition and clearance of pathogens [8, 9]. AMϕ are also at the center of ALI pathogenesis through their secretion of cytokines and chemokines that regulate lung inflammation in response to pulmonary infection [8]. A recent study from our laboratory showed that exosomes released from hemorrhagic shock-activated AMϕ induced the production of NADPH oxidase-derived reactive oxygen species (ROS) inside PMN, resulting in necroptosis and subsequently enhancing lung inflammation [7].

Exosomes are extracellular vesicles (EVs) with lipid bilayer membranes, and they range in size from 30 to 100 nm. Exosomes are formed as intraluminal vesicles (ILVs) by budding into early endosomes to form multivesicular bodies (MVBs) and can contain miRNA, proteins, and lipids [10]. The biogenesis of ILVs involves endosomal sorting complexes required for transport (ESCRT) machinery, lipids and tetraspanins. The transport of MVBs to the plasma membrane is a critical step for exosome secretion [11, 12] and involves important roles for Rab proteins, such as Rab11, Rab27 and Rab35 [13–15]. Studies have indicated that Rab27a and Rab27b control the steps that the vesicles undergo during docking to their target compartment, which leads to vesicular fusion with the plasma membrane and the subsequent secretion of CD63-containing exosomes [14].

Alveolar epithelial cells form a barrier serving as the first line of pathogen defense in the alveolus, and they are essential for maintaining lung respiratory function [16]. The alveolar epithelium comprises two main cell types: alveolar type I cells and alveolar type II cells. Type I cells mainly form the gas exchange surface in the alveolus, whereas type II cells have many important metabolic and biosynthetic functions, including the synthesis and secretion of the surfactant, which is a lipid-protein complex. Alveolar type II cells are also considered to be progenitors of the alveolar epithelium because of their ability to both proliferate and differentiate into alveolar type I cells. Recent studies have recognized important roles for type II cell-derived cytokines, including interleukin (IL)-25, IL-33, and thymic stromal lymphopoietin (TSLP), in the regulation of lung inflammation [17, 18].

In this study, we explore a novel function of LEPCs in regulating the release of exosomes from AMϕ following LPS stimulation. We demonstrate that LPS induces macrophages (Mϕ) to release exosomes, which are internalized by neighboring Mϕ to promote TNF-α expression. The secreted IL-25 by LEPCs downregulates Rab27a and Rab27b expression in Mϕ, thereby suppressing both exosome release from Mϕ and TNF-α expression and secretion. These findings reveal a previously unidentified crosstalk pathway between LEPCs and Mϕ that negatively regulates the inflammatory responses of Mϕ to LPS. Modulating IL-25 signaling and targeting exosome release may present a new therapeutic strategy for the treatment of ALI.

## Methods

### Animal strains

Eight-week-old C57BL/6 wildtype (WT) male mice were purchased from The Jackson Laboratory (Bar Harbor, ME, USA). TLR4 knockout (TLR4^−/−^) mice were bred in Dr. Billiar’s laboratory at the University of Pittsburgh. All animal experimental protocols were reviewed and approved by the Institutional Animal Care and Use Committees of the University of Pittsburgh and the VA Pittsburgh Healthcare System. The mice were given food and water ad libitum.

### Exosome isolation and characterization

Exosomes were isolated from culture supernatants of bone marrow-derived macrophages (BMDM) maintained in serum-free DMEM with 1% penicillin/streptomycin. Supernatants from cultured BMDM were collected and centrifuged at 2000 g for 30 min to remove debris, followed by the addition of Total Exosome Isolation Reagent (ThermoFisher Scientific, Pittsburgh, PA) and incubation at 4 °C overnight following the manufacturer’s instructions. The mixture was centrifuged at 10,000 g for 60 min at 4 °C. The final pellet containing the exosomes was resuspended in PBS. To detect exosome marker proteins, 100 μl of exosomes isolated from medium or BALF were incubated with 10 μl of aldehyde/sulfate latex beads (4 μm diameter, Life Technologies, Grand Island, NY) for 15 min at 4 °C. PBS was then added to the exosomes to increase the total volume to 400 μl, followed by overnight incubation at 4 °C with gentle agitation. The exosome-coated beads were stained with PE-conjugated anti-mouse CD63 antibody for 1 h at room temperature and were analyzed by flow cytometry.

### BMDM isolation and culture

BMDM were cultured as in our previous studies [19, 20]. Briefly, bone marrow from femurs and tibias harvested from mice was flushed with prechilled DMEM. The cell pellets were collected, and the erythrocytes were lysed with RBC lysis buffer. The resulting cells were suspended in BMDM culture medium (DMEM containing 10% FBS supplemented with 50 μg/ml penicillin/streptomycin and 10 ng/ml recombinant macrophage colony-stimulating factor [M-CSF; Thermo Fisher Scientific]) at a concentration of 1 × 10^6^ cells/ml and seeded into 6-well plates. The culture medium for BMDM was changed on day 3 and day 5. The BMDM were fully differentiated and ready for use on day 7.

### Mouse LEPC isolation

Mice were euthanized with an overdose of pentobarbital (50 mg/kg BW). The lungs were perfused with 10 ml PBS through the right ventricle of the heart, and the lung tissue was then diced into pieces of approximately 1 mm^3^ for digestion in 5 ml of digestion medium containing DMEM/F12 with collagenase/dispase for 30–45 min at 37 °C with vortexing every 10 min. The resulting samples were homogenized with 70 μm cell strainers, and the cell pellet was then collected and treated with DNase I (1 μg/ml) for 10 min. The remaining red blood cells were lysed with RBC lysis buffer (Life Technologies Corporation, Grand Island, NY) The cells were labeled with biotin-conjugated anti-mouse CD326 antibody and then incubated with streptavidin-conjugated immunomagnetic beads for 1 h at 4 °C. The CD326-positive epithelial cells were selected by flow cytometry and cultured in DMEM/F12 supplemented with 10% FBS and 50 μg/ml penicillin/streptomycin.

### LEPC and BMDM coculture

LEPCs and BMDM were cocultured using the Transwell™ system. LEPCs (1 × 10^6^ cells per well) were seeded in the 6-well Transwell™ inserts, and BMDM (1 × 10^6^ cells per well) were seeded in the 6-well plates. After coculture, the supernatant was harvested for further analysis.

### Intratracheal injection of LPS in mice

Mice were anesthetized with ketamine (50 mg/kg B.W.) combined with xylazine (5 mg/kg B.W.). LPS (2 mg/kg B.W.) in 0.2 ml of saline was delivered via intratracheal aerosol administration using a MicroSprayer® aerosolizer high-pressure syringe (Penn-Century, Wyndmoor, USA). The sham animals underwent the same anesthetic procedure and the intratracheal aerosol injection of 0.2 ml of saline. Bronchoalveolar lavage fluid (BALF) was collected, and the AMϕ were isolated for further analysis.

### Flow cytometry

BMDM-derived exosomes bound to aldehyde/sulfate latex beads were stained with PE-CD63 (exosome maker) or PE-isotype control antibody, followed by analysis with a BD FACS flow cytometer. Control and LPS-treated BMDM or AMϕ were stained with PE-IL25R or PE-isotype control antibody followed by flow cytometric analysis. The mean fluorescence intensity (MFI) was calculated by FlowJo v10.0.

### Confocal immunofluorescence

Exosomes were isolated from the supernatant of control or LPS-treated BMDM and stained with DiI cell-labeling solution at 37 °C for 20 min. The DiI-labeled exosomes were incubated with BMDM at 37 °C for 2 h. The cells were fixed with 4% paraformaldehyde for 15 min at room temperature. The nuclei were counterstained with Hoechst 33,258. Fluorescence images were captured by confocal microscopy.

### Western blotting

BMDM lysates were separated by 12% SDS-PAGE and transferred onto PVDF membranes. After incubation for 1 h at room temperature with blocking buffer (LI-COR Biosciences, Lincoln, NE, USA), blots were incubated with a primary antibody (Rab27a, Rab27b, or GAPDH) at 4 °C overnight followed by incubation with an appropriate secondary antibody (LI-COR Biosciences) for 1 h at room temperature. Protein bands were detected using the Odyssey system from LI-COR Biosciences and were quantified using Image J version 1.50i.

### RNA extraction and quantitative real-time PCR

The plated cells were harvested, and total RNA was isolated using TRIzol® RNA isolation reagents (Thermo Fisher Scientific, Pittsburgh, PA, USA) following the manufacturer’s instructions. Reverse transcription was performed using iScript™ Reverse Transcription Supermix (170–8840, Bio-Rad) following the manufacturer’s instructions. Real-time RT-PCR was performed using i*Taq*™ Universal SYBR® Green Supermix (1,725,121, Bio-Rad) in a Bio-Rad iQ5 real-time PCR system (Bio-Rad). The following gene-specific primers were used for gene amplification: *TNFa* forward, 5’-GACGTGGAACTGGCAGAAG-3′ and reverse, 5′-TTGGTGGTTTGTGAGTGTG-3′; *IL6* forward, 5’-CCAAGAGGTGAGTGCTTCCC-3′ and reverse, 5’-CTGTTGTTCAGACTCTCTCCCT-3′; and *18S* forward, 5’-GTAACCCGTTGAACCCCATT-3′ and reverse, 5’-CCATCCAATCGGTAGTAGCG-3′. Amplification was performed with cycling conditions of 15 s at 95 °C followed by 30 s at 60 °C for 40 cycles. After the amplification protocol was completed, the PCR product was subjected to melting curve analysis using the Bio-Rad iQ5 software (Bio-Rad). The fold change was calculated using the ΔΔ threshold cycle method, and the value for the 18S rRNA gene was used to normalize the gene expression in the experimental groups to that in the untreated groups.

### Reagents

PE-conjugated anti-mouse CD63 antibody (143903); PE-conjugated rat IgG2a, κ isotype ctrl antibody (400507); anti-mouse IL-25 (IL-17E) antibody (514403); and purified Rat IgG1, κ Isotype control antibody (400413) were from Biolegend (San Diego, CA, USA). Exosome-releasing inhibitor DMA (sc-202,459) was purchased from Santa Cruz (Dallas, TX, USA). LPS (L2880), and polymyxin B-agarose (P1411) and DNase I (11284932001) were purchased from Sigma-Aldrich (St. Louis, MO, USA). Recombinant mouse IL-17E (IL-25) protein (1399-IL-025), recombinant mouse IL-33 protein (3632-ML), and recombinant mouse TSLP protein (555-TS) were purchased from R&D Systems. The IL-25 mouse ELISA kit (88–7002-22), TNF alpha mouse ELISA kit (88–7324-86), DiI cell-labeling solutions (V22885), total exosome isolation reagent (4478359), PE-IL-25R (IL-17RB) monoclonal antibody (MUNC33) (12–7361-80), PE-Cyanine7 CD326 (EpCAM) monoclonal antibody (1B7), biotin-conjugated CD326 (13–5791-82), and aldehyde/sulfate latex beads (4% *w*/*v*, 4 μm diameter, A37304) were purchased from Thermo Fisher Scientific (Pittsburgh, PA, USA). Anti-Rab27a (69295S) and anti-GAPDH (5174S) antibody were from Cell Signaling Technology (Danvers, MA, USA). Anti-Rab27b (ABS1026) was from MilliporeSigma (Kankakee, IL, USA). The siNC, siRab27a, and siRab27b RNAs were purchased from Integrated DNA Technologies (Coralville, IA, USA). BD IMag™ Streptavidin Particles Plus were purchased from BD Biosciences (San Jose, CA, USA).

### Statistical analysis

The data are presented as the mean ± SEM of the indicated number of experiments/repeats. SPSS 20.0 or GraphPad Prism v.6.0 was used for statistical analysis. Significance differences between groups were determined by one-way ANOVA, two-way ANOVA, or independent sample two-tailed Student’s *t*-test, and *P* < 0.05 was considered statistically significant.

## Results

### LEPCs suppress the LPS-induced release of exosomes from Mϕ

**Fig. 1:**
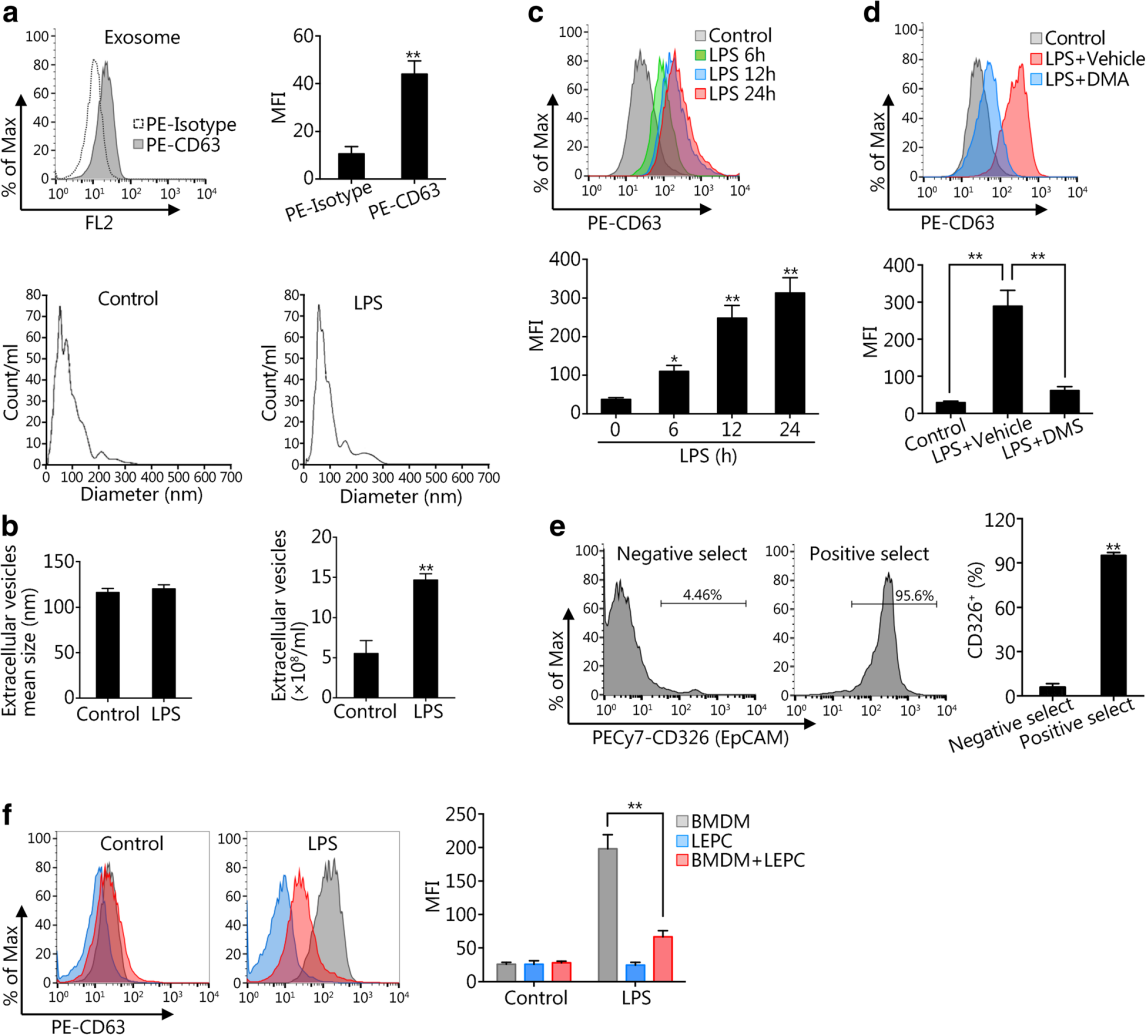
LEPCs suppress LPS-induced exosome release from Mϕ. **a** Exosomes were isolated from BMDM cultured in serum-free medium, stained with PE-isotype and PE-CD63 antibodies and measured by flow cytometry. **b** Exosomes isolated from the culture media of control and LPS-treated BMDM were analyzed for the mean particle diameter and total number by NanoSight. **c** BMDM were treated with LPS (1 μg/ml) for 0, 6, 12, and 24 h. Exosomes were then isolated from the culture medium and detected by CD63 staining and flow cytometry. **d** BMDM were treated with LPS (1 μg/ml) in the presence or absence of dimethyl amiloride (DMA, 25 μM) for 24 h. Exosomes were then isolated from the culture medium and detected by CD63 staining and flow cytometry. **e** Lung cells were labeled with biotin-conjugated CD326 antibody and separated with streptavidin-conjugated immunomagnetic beads. Flow cytometric analysis shows a purity of greater than 95% for the yielded LEPCs. **f** BMDM were cultured alone or cocultured with LEPCs and were either treated with LPS (1 μg/ml) for 24 h or not treated. Exosomes were isolated from the culture medium and detected by CD63 staining and flow cytometry. All results are representative of at least three independent experiments. The graphs show the mean ± SEM, *n* = 3; *P < 0.05 or **P < 0.01, compared with the indicated groups

BMDM were cultured without serum for 24 h, and exosomes released from BMDM were isolated and identified by staining with the exosome marker CD63 and analysis by flow cytometry as shown in Fig. 1a. In addition, nanoparticle tracking analysis using NanoSight was used to determine the diameter and number of the extracellular particles in the culture media of BMDM with or without 24 h of LPS treatment. The diameter of the extracellular particles in both groups was in the range of ~ 100 nm (Fig. 1b), suggesting that the particles were exosomes; however, the total number of extracellular vesicles in the LPS-treated group was significantly increased (Fig. 1b). LPS treatment of BMDM for up to 24 h significantly increased the release of exosomes from Mϕ in a time-dependent manner (Fig. 1c), and pretreatment of BMDM with the exosome release inhibitor dimethyl amiloride (DMA, 25 μg/ml) [21] prevented the LPS-induced release of exosomes (Fig. 1d).

To study the interaction between lung epithelial cells (LEPCs) and macrophages, CD326-positive LEPCs, which are mainly type II epithelial cells [22, 23], were isolated from mouse lung tissue using magnetic-activated cell sorting (MACS) [22, 24] and confirmed by flow cytometry (Fig. 1e). The results showed that the selection of CD326^+^ LEPCs reached a purity of > 95%. We further cocultured LEPCs with BMDM using Transwell plates and treated the cultures with LPS for 24 h. Interestingly, the exosome release from monocultured LEPCs was very low in both the LPS-treated and non-LPS-treated conditions (Fig. 1f). We also found that the exosome release in response to LPS in the cocultured group was significantly decreased compared with that in the BMDM monoculture group (Fig. 1f). These results suggest that the release of macrophage-derived exosomes is suppressed by LEPCs.

### IL-25 mediates LEPC suppression of macrophage-derived exosome release

**Fig. 2:**
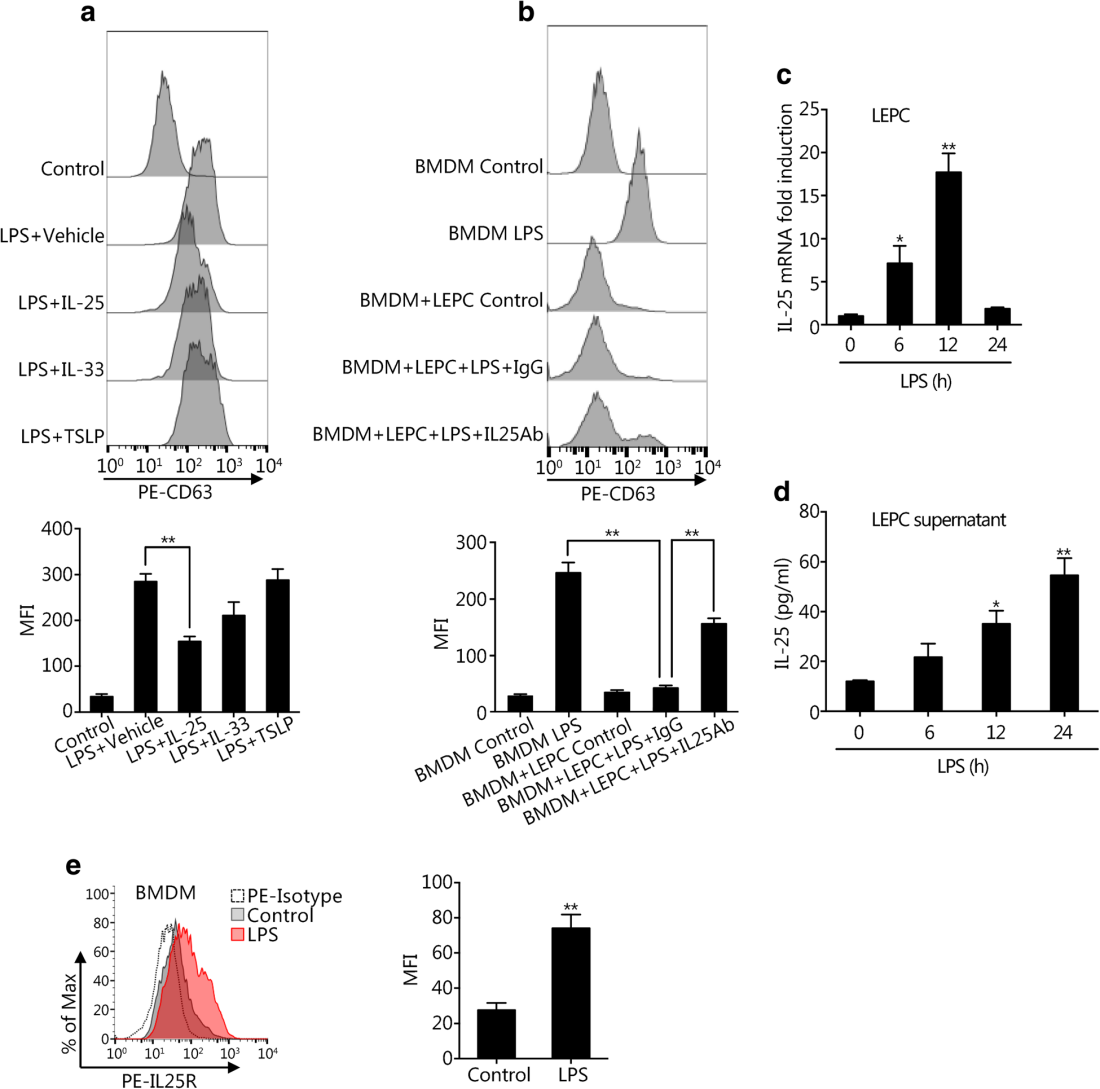
IL-25 mediates LEPC-mediated suppression of exosome release from Mϕ. **a** BMDM were treated with LPS (1 μg/ml) with or without recombinant IL-25 (200 ng/ml), IL-33 (200 ng/ml), or TSLP (200 ng/ml) for 24 h followed by isolation of exosomes from the cell culture supernatants, and CD63+ exosomes were identified by flow cytometry. **b** BMDM cultured alone or cocultured with LEPCs were treated with LPS (1 μg/ml) in the presence or absence of anti-IL-25 neutralizing antibody (10 μg/ml) or control non-specific IgG (10 μg/ml). CD63+ exosomes were detected by flow cytometry. **c** and **d** LEPCs were treated with LPS (1 μg/ml) for 0, 6, 12, or 24 h, and the IL-25 mRNA expression in the LEPCs and IL-25 protein concentration in the supernatants were measured by RT-qPCR (**c**) and ELISA (**d**), respectively. **e** BMDM were treated with LPS (1 μg/ml) for 24 h, and the cell surface expression of IL-25R was measured by flow cytometry. All results are representative of three independent experiments. The graphs show the mean ± SEM, *n* = 3; *P < 0.05 or **P < 0.01, compared with the indicated groups or with the control

LEPCs release innate cytokines, such as TSLP, IL-33, and IL-25, in response to infection or various environmental factors [17, 18, 25]. To determine whether the suppression of macrophage-derived exosome release by LEPCs is mediated through epithelial cytokines, we treated BMDM with LPS and recombinant TSLP, IL-25, or IL-33 for 24 h. As shown in Fig. 2a, recombinant IL-25 exhibited a significant suppressive effect on the LPS-induced release of exosomes from BMDM, whereas TSLP and IL-33 did not significantly affect exosome release. Furthermore, compared to the addition of non-specific IgG, the addition of anti-IL-25 neutralizing antibody to the LPS-treated LEPC-BMDM coculture system partially restored the release of exosomes from BMDM (Fig. 2b).

To confirm whether IL-25 was secreted from LEPCs in response to LPS, we measured IL-25 mRNA expression in LEPCs and IL-25 concentration in the culture supernatant following LPS treatment for up to 24 h. As shown in Fig. 2c, IL-25 mRNA expression increased in LEPCs following LPS treatment and reached a peak at 12 h after LPS treatment. The IL-25 protein concentration in the culture supernatant of LEPCs also increased and reached a peak at 24 h after LPS treatment (Fig. 2d). We further found that IL-25 receptor (IL-25R) expression in BMDM increased in response to LPS treatment (Fig. 2e). These results suggest enhanced IL-25-induced signaling in macrophages.

### IL-25 downregulates LPS-induced Rab27a and Rab27b expression in Mϕ and suppresses exosome release

**Fig. 3:**
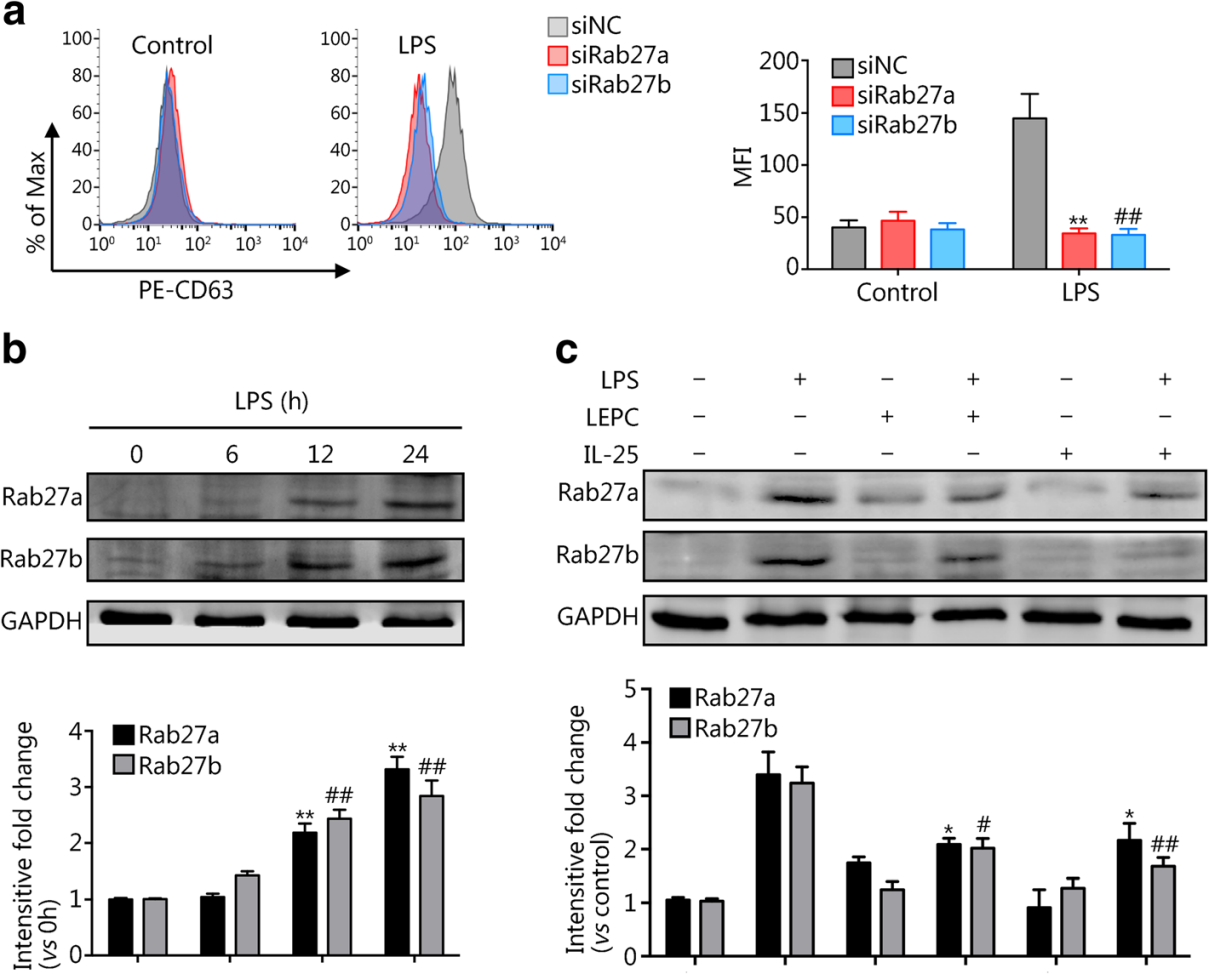
IL-25 downregulates LPS-induced Rab27a and Rab27b to suppress exosome release from macrophages. **a** BMDM were transfected with siRab27a, siRab27b, or siNC (control) for 36 h and were then treated with LPS (1 μg/ml) for 24 h. Exosomes were isolated from the culture media, and CD63 staining was detected by flow cytometry. **b** BMDM were treated with LPS (1 μg/ml) for 0, 6, 12, and 24 h. Rab27a and Rab27b expression were detected by Western blotting. **c** BMDM cultured alone or cocultured with LEPCs were treated with LPS (1 μg/ml) in the presence or absence of recombinant IL-25 (200 ng/ml) for 24 h. The expression of Rab27a and Rab27b were detected by Western blotting. All results are representative of three independent experiments. The graphs show the mean ± SEM, *n* = 3; * or # *P* < 0.05, ** or ## *P* < 0.01, compared with the LPS groups. * and ** indicate Rab27a, # and ## indicate Rab27b

The Ras-related proteins Rab27a and Rab27b have been reported to play a role in multivesicular endosome docking at the plasma membrane; and are therefore important in regulating exosome secretion [11, 14]. To elucidate whether LEPC-derived IL-25 affects Rab27a and Rab27b to suppress exosome release from macrophages, we first measured Rab27a and Rab27b expression in BMDM following LPS treatment. The expression of both Rab27a and Rab27b increased in BMDM starting at 12 h after LPS stimulation (Fig. 3b), and coincubation with LEPCs or treatment with IL-25 markedly decreased Rab27a and Rab27b expression in BMDM at 12 h after LPS treatment (Fig. 3c). Furthermore, siRNA knockdown of Rab27a and Rab27b in BMDM significantly decreased exosome release from the macrophages in response to LPS stimulation (Fig. 3a). These data suggest that IL-25 downregulates LPS-induced Rab27a and Rab27b expression to suppress exosome release from macrophages.

### Suppression of exosome release from Mϕ attenuates TNFα secretion by Mϕ

**Fig. 4:**
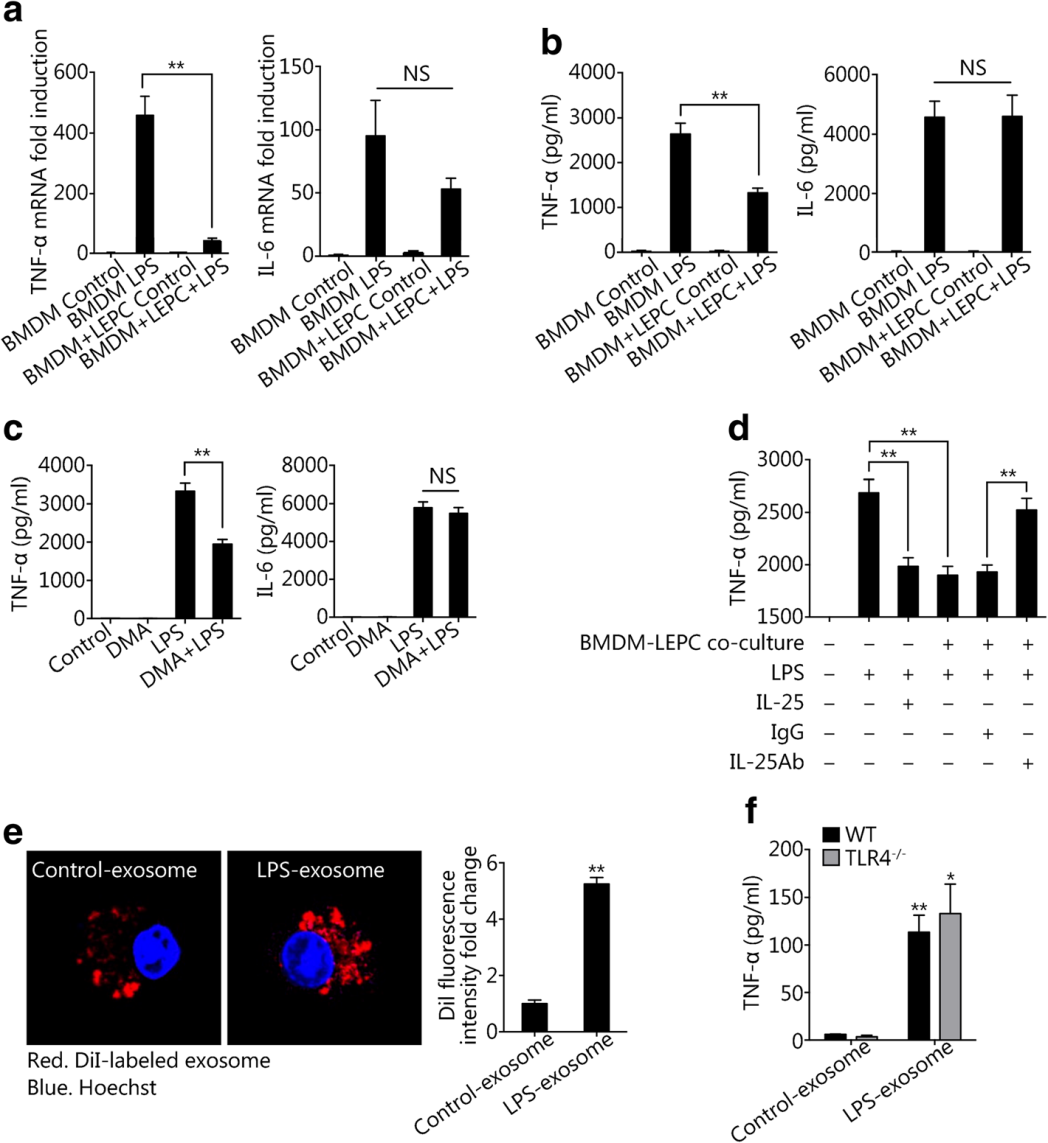
Suppression of exosome release from Mϕ attenuates the secretion of TNFα from Mϕ. **a** and **b** BMDM cultured alone or cocultured with LEPCs were treated with LPS (1 μg/ml) for 24 h. Then, TNF-α and IL-6 mRNA levels in the BMDM and protein levels in the supernatants were measured by RT-qPCR and ELISA, respectively. **c** BMDM were treated with LPS (1 μg/ml) with or without dimethyl amiloride (DMA, 25 μmol/L) for 24 h, and then the TNF-α and IL-6 levels in the supernatants were measured by ELISA. **d** BMDM cultured alone or cocultured with LEPCs were treated with LPS (1 μg/ml) in the presence or absence of recombinant IL-25 (200 ng/ml), anti-IL-25 neutralizing antibody (10 μg/ml), or non-specific IgG (10 μg/ml) for 24 h. The level of TNF-α in the supernatants was measured by ELISA. **e** Immunofluorescence images show Mϕ internalization of exosomes. BMDM were incubated with DiI-labeled exosomes (red) that were isolated from the culture media of untreated or LPS-treated BMDM (1 μl/ml) for 2 h. Nuclei were counterstained with Hoechst (blue). The fold changes in DiI fluorescence intensity were calculated by ImageJ. **f** Exosomes were isolated from the culture media of untreated or LPS-treated BMDM (1 μl/ml) for 24 h and were then added to WT or TLR4^−/−^ BMDM and incubated for 6 h. The level of TNF-α in the supernatants was measured by ELISA. All results are representative of three independent experiments. Three random fields in the images were counted. The graphs show the mean ± SEM, *n* = 3; *P < 0.05 or **P < 0.01, compared with the indicated groups or with the control. NS, no significant difference

To investigate the influence of suppressed exosome release on the development of inflammation, we measured alterations in the expression of TNF-α and IL-6 mRNA and protein in BMDM. As shown in Fig. 4a and b, coculture of LEPCs and BMDM resulted in significantly decreased levels of TNF-α mRNA and protein in BMDM at 24 h after LPS treatment. However, the IL-6 mRNA and protein levels in these BMDM did not change significantly. This observation led us to hypothesize that macrophage-derived exosomes may augment the expression of TNF-α in macrophages through autocrine or paracrine mechanisms. To test this hypothesis, we blocked exosome release using DMA to inhibit exosome release-related signaling. As shown in Fig. 4c, the blockade of exosome release from Mϕ significantly decreased the expression of TNF-α but not of IL-6 in Mϕ at 24 h after LPS treatment. Pretreatment with recombinant IL-25, which, as we showed above, suppressed exosome release from Mϕ, decreased LPS-induced TNF-α expression in Mϕ. Conversely, treatment with anti-IL-25 neutralizing antibody reversed the suppression of TNF-α expression in BMDM cocultured with LEPCs (Fig. 4d).

To determine whether exosomes derived from Mϕ are able to enter or reenter macrophages, we isolated exosomes from the culture supernatants of control and LPS-treated BMDM and labeled them with DiI. We then added the labeled exosomes to unstimulated BMDM for 2 h and assessed internalization by confocal immunofluorescence. We observed internalization of exosomes from both unstimulated and LPS-stimulated Mϕ in the culture of BMDM (Fig. 4e). However, significantly more exosomes from LPS-treated Mϕ were internalized compared to the number of internalized exosomes from unstimulated Mϕ (Fig. 4e).

To address whether LPS in exosomes contributes to exosome-stimulated increases in TNF-α expression in Mϕ, we isolated exosomes from the culture supernatants of control and LPS-treated BMDM and treated them with polymyxin B (PMB), which binds the lipid A moiety of LPS and neutralizes the biological effects of LPS [26]. We then treated BMDM isolated from WT and TLR4^−/−^ mice with the control and LPS-treated exosomes +/− PMB for 6 h. As shown in Fig. 4f, PMB-treated exosomes from LPS-induced BMDM significantly increased TNF-α expression in WT and TLR4^−/−^ BMDM. These results suggest that components of LPS-induced exosomes but not the contaminating LPS are responsible for increasing TNF-α expression in Mϕ. .

### IL-25 suppresses AMϕ exosome release in vivo

**Fig. 5:**
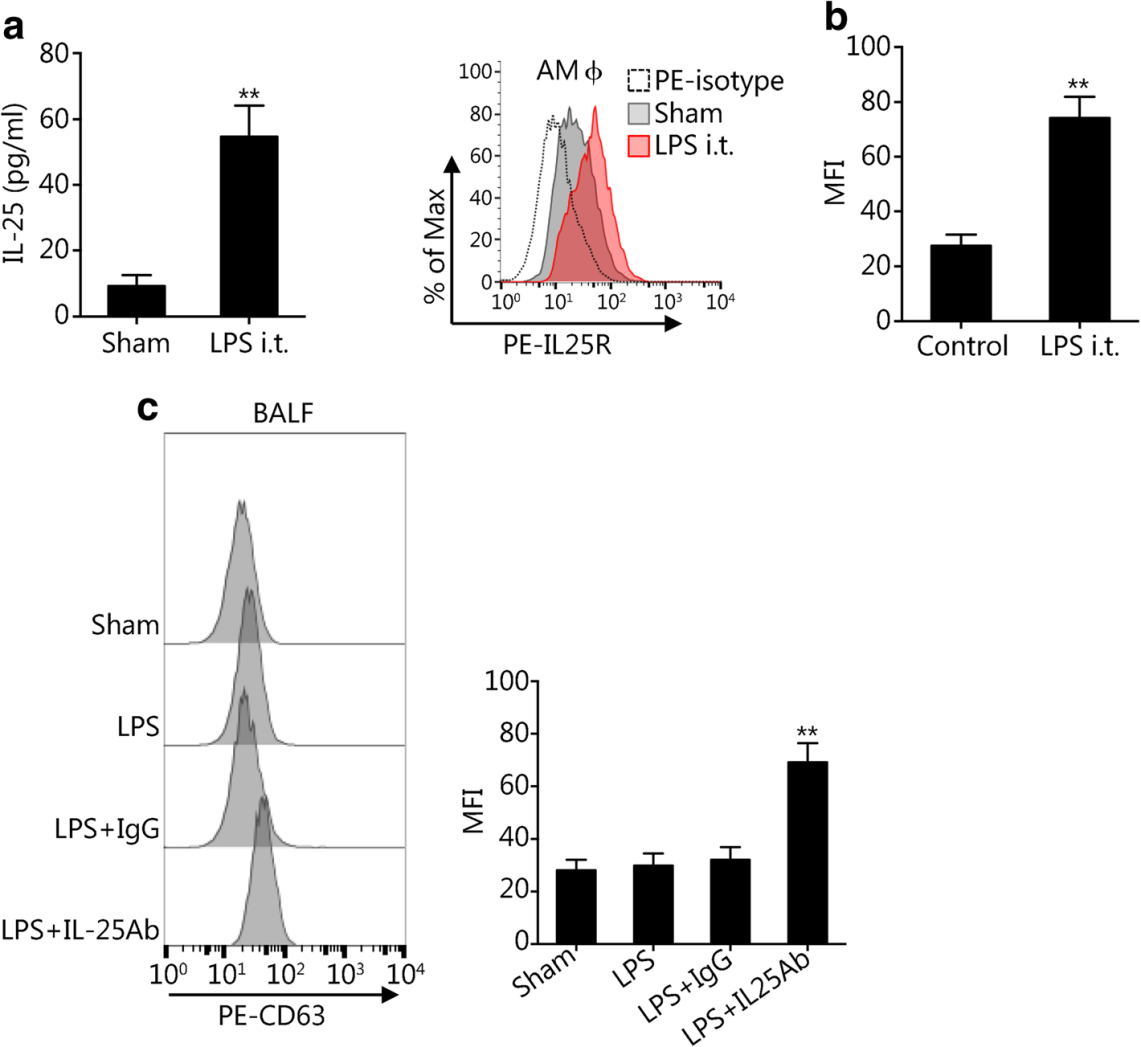
IL-25 suppresses exosome release from AMϕ in vivo. **a** and **b** WT mice were given intratracheal (i.t.) LPS (2 mg/kg BW in a volume of 100 μl/mouse) or sham (i.t. PBS, 100 μl/mouse) for 24 h, and BALF was collected. IL-25 was measured in the supernatant by ELISA (**a**) and the surface expression of IL-25R on AMϕ was detected by flow cytometry (**b**). **c** WT mice were treated with LPS (2 mg/kg BW in a volume of 100 μl/mouse, i.t.), sham (PBS, 100 μl/mouse, i.t.), LPS (2 mg/kg BW, i.t.) + IgG (1 mg/kg BW, i.t.); or LPS (2 mg/kg BW, i.t.) + anti-IL-25 antibody (1 mg/kg BW, i.t.) for 24 h. Exosomes were isolated from BALF and quantified by CD63 staining and flow cytometry. All results are representative of three independent experiments. The graphs show the mean ± SEM, *n* = 3; *P < 0.05 or **P < 0.01, compared with the indicated groups or with the control

To recapitulate the in vitro study in vivo, we treated WT mice with intratracheal (i.t.) injections of LPS to induce acute lung injury. At 24 h after i.t. LPS administration, the IL-25 concentration in the bronchoalveolar lavage fluid (BALF) was significantly increased compared to the concentration in the BALF of sham mice (Fig. 5a). The IL-25R surface expression on AMϕ was also markedly increased in LPS-treated mice compared to sham mice (Fig. 5b). Notably, anti-IL-25 neutralizing antibody increased exosome release in response to i.t. LPS (Fig. 5c). Collectively, these results suggest an important role for LEPC-derived IL-25 in suppressing the secretion of exosomes from AM in response to LPS.

## Discussion

ALI is a common and severe complication following pulmonary infection [27]. The activation of innate immunity is critically involved in the progression of ALI. In this study, we identified a novel mechanism by which LEPCs negatively regulate exosome release from Mϕ through IL-25 in response to the bacterial product LPS. Our results show that LEPC-derived IL-25 downregulates Rab27a and Rab27b expression in Mϕ and subsequently suppresses exosome release from Mϕ and attenuates exosome-induced TNF-α expression and secretion from Mϕ.

Our previous studies revealed roles for cell-cell interactions between multiple cell types in the progression of ALI. We have shown that interactions between PMN and endothelial cells contribute to the enhanced expression of ICAM-1 on endothelial cells and subsequently augment the adhesion and transmembrane migration of PMN into infected lungs [28]. We also found that AMϕ are a major regulatory cell population that can actively influence the function of other cell populations. For example, we reported that chemokines derived from AMϕ are a determinant of the migration of PMN through the regulation of G-protein coupled receptor kinase expression in PMN and the surface expression of chemokine receptors on PMN [7, 29]. Our recent study showed that exosomes released from AMϕ act as an important mediator to induce necroptosis of PMN and enhance lung inflammation following hemorrhagic shock [7]. The current study provides further evidence to support important roles for cell-cell interactions in the regulation of lung inflammation. More importantly, this study shows that the function of AMϕ can also be regulated by other cell populations such as LEPCs.

The results demonstrate an important role for LEPC-derived IL-25 in regulating the release of exosomes from macrophages. We were unable to detect IL-25 in supernatants from BMDM after LPS stimulation. However, the sensitivity of the ELISA is a limitation, and we may not be able to exclude the possibility that BMDM release IL-25 in response to LPS. Nonetheless, the data shown in Fig. 2a, b, c and d support the conclusion that LEPCs are the major source of IL-25 that affects the release of exosomes from macrophages. Notably, as shown in Fig. 2b, exosome release from LPS-stimulated BMDM did not return to its peak level after IL-25 was blocked with a neutralizing antibody in the co-culture of LEPCs with BMDM. We are aware that the binding of antibody to antigen follows the rule of binding dynamics involving a disassociation constant, and the achievement of > 90% antigen inhibition requires a very high concentration of the neutralizing antibody. Therefore, we believe that the antibody concentration used in the experiments may not have reached a level that was able to completely abolish the effects of IL-25; thus, the MFI of the “BMDM +LEPC+LPS + IL25Ab” group did not exhibit 100% restoration. However, the difference in MFI between the “BMDM+LEPC+LPS + IgG” and “BMDM+LEPC+LPS + IL25Ab” groups (Fig. 2b) should clearly reflect the role of IL-25 in suppressing exosome release from BMDM.

Exosome secretion requires the fusion of multivesicular bodies (MVBs) with the cell plasma membrane. The Rab GTPases critically regulate the multiple steps of membrane trafficking, including vesicle budding, vesicle transport, and membrane fusion. It has been reported that knockdown of Rab family members, including Rab2b, Rab5a, Rab9a, Rab27a, or Rab27b, significantly decreases exosome secretion [30, 31]. Rab27a and Rab27b have been reported as important regulatory factors governing intracellular vesicular trafficking critical for MVB docking to the plasma membrane [14]. In this study, we also demonstrated an important role for Rab27a and Rab27b in the regulation of the release of exosomes from Mϕ. This role is evidenced by the following observations: 1) Mϕ Rab27a and Rab27b expression increased in response to LPS; 2) knockdown of Rab27a or Rab27b significantly decreased exosome release from Mϕ; and 3) downregulation of LPS-induced Rab27a and Rab27b expression by IL-25 suppressed exosome release from Mϕ. Interestingly, our study identifies a novel role for IL-25 in downregulating the expression of Rab27a and Rab27b in Mϕ, although the mechanism underlying the IL-25-mediated regulation of Rab27a and Rab27b expression is unknown, and future studies will be needed to elucidate the signaling process and mechanism.

Cytokines, chemokines and cell surface receptors are well known to mediate intercellular communication. Emerging evidence suggests that exosomes also serve as important mediators of cell-cell interaction [7]. Exosomes carry a variety of different molecules that can be taken up by recipient cells [32]. In this study, we found that exosomes derived from Mϕ are important mediators that promote the expression of TNF-α in Mϕ. By tracking exosomes with DiI staining, we observed that extracellular exosomes were internalized by Mϕ, which further promoted the expression of TNF-α but not of IL-6. These results suggest a specific pathway mediating exosome-induced TNF-α expression, although at present, it is not clear which exosome component is responsible. LPS contamination of exosomes does not appear to be the main stimulator, since neither PMB treatment of exosomes nor TLR4 knockout in Mϕ reduced exosome-induced TNF-α expression.

In summary, our study demonstrates a novel mechanism underlying the crosstalk between LEPCs and Mϕ and its potential role in the regulation of ALI. Modulating IL-25 signaling and targeting exosome release may present new therapeutic strategies for the treatment of ALI.

## Conclusions

This study demonstrates that LPS induces macrophages (Mϕ) to release exosomes, which are then internalized by neighboring Mϕ to promote TNF-α expression. The IL-25 secreted from LEPCs downregulates Rab27a and Rab27b expression in Mϕ, resulting in suppressed exosome release and thereby attenuating exosome-induced TNF-α expression and secretion. These findings reveal a previously unidentified pathway of crosstalk between LEPCs and Mϕ that negatively regulates inflammatory responses of Mϕ to LPS. Modulating IL-25 signaling and targeting exosome release may present a new therapeutic strategy for the treatment of ALI.

## Abbreviations

ALI: Acute lung injury
AMϕ: Alveolar macrophages
ARDS: Acute respiratory distress syndrome
BALF: Bronchoalveolar lavage fluid
BMDM: Bone marrow derived-macrophages
DMA: Dimethyl amiloride
i.t.: Intratracheal
IL: Interleukin
LEPC: Lung epithelial cells
LPS: Lipopolysaccharide
MVB: Multivesicular bodies
Mϕ: Macrophages
PMB: Polymyxin B
TLR4: Toll-like receptor 4
TSLP: Thymic stromal lymphopoietin
